# Supplementary material for: Head movements affect skill acquisition for ball trapping in blind football
Source: PLoS One. 2024 Nov 7;19(11):e0313336. doi: 10.1371/journal.pone.0313336 (PMC11542860; doi:10.1371/journal.pone.0313336)
Supplement: Attachment — Submitted filename: Response_to_Reviewers.docx [file pone.0313336.s003.docx]

**Comments from** **Editor:**

**General Comment:** *Thank you for submitting your manuscript to PLOS ONE. After careful consideration, we feel that it has merit but does not fully meet PLOS ONE’s publication criteria as it currently stands. Therefore, we invite you to submit a revised version of the manuscript that addresses the points raised during the review process.*

*An external reviewer and myself reviewed your manuscript. Please carefully address the comments below and pay particular attention to some methodological points, some of which being also highly related to the motivation and hypotheses of the stud (e.g., definition of variables and relevance of these variables to the hypotheses).*

**Response:**

We wish to express our strong appreciation to the Editor for your constructive and insightful comments on our paper. The comments have helped us significantly improve the paper. We have carefully considered the editor’s comments and incorporated changes that reflect the detailed suggestions you have generously provided.

Please see below our detailed response to each comment where we also describe the changes made to the manuscript. All page numbers refer to the revised manuscript file with tracked changes.

**1. Comment:** [*At the moment there are several instances that require justificaiton and clarification. For instance, what exactly is meant by "performance" (e.g., line 179) and how is this calculated? I think these refer to the absolute and variable errors, but you should explain in detail how these were calculated. In addition, again regarding the AE and VE, it is unclear how these variables are related to the research question and what are the underlying hypotheses. This needs to be sharpened so that the conducted statistical tests can be justified.*]

**Response:** We appreciate the editor’s instructive suggestion. Following this suggestion, we have explained in detail how the absolute and variable errors were calculated (p. 10, lines 213-219). We have also added a description to clarify the research question regarding these variables and the underlying hypotheses (p. 5, lines 95-100).

**2. Comment:** [*Another major point related to the same topic is the experimental manipulations of distance and speed. First, although the factor "distance" is* *included in the statistics, the factor "speed" is not. Why is "speed" a factor in the experiment but not in the statistics? Are there no hypotheses about this? Likewise, what are the hypotheses about the effect of "distance"? This needs to be explicitly stated in the Introduction. As far as I understand, the factor "direction" is not included in the design either. Please explain the methodological decision about the conditions, the underlying hypotheses and justify the statistical testing employed.*]

**Response:** We thank you for pointing this out. We have added a description in the *Introduction* to more clearly explain our methodological decision about the factor ‘distance’ (p. 5-6, lines 101-107) and the underlying hypotheses (p. 6, lines 108-112). As the editor suggests, the factor ‘speed’ is not included in the statistics in our study. We have now described the reason for this based on the previous study. Specifically, the ball speed was randomised to prevent participants from predicting the timing of the ball's approach, which could otherwise affect their performance (p. 8, lines 157-161).

**3. Comment:** [*A last major comment is about the comparisons across the timecourse. How is inflation of type I error being compensated here? I am also not confident that the description of the related analysis as well as the underlying hypotheses are explicit enough. Please revise accordingly.*]

**Response:** We thank the editor for pointing out the comparisons across the time course. Following the editor’s comment, we have added a description of the related analysis, including Bonferroni correction for multiple comparisons (p. 10, lines 209-213 and p. 11, lines 230-232) as well as the underlying hypotheses (p. 5 lines 84-88 and p. 6 lines 110-112). We have also revised the corresponding results (p. 16, lines 319–321) and the legends for Figure 5 (p.17, lines 334-337) and Table 3 (p. 19, lines 341-343).

**4. Comment:** [*Line 101, Declaration of Helsinki: please mention which version you are referring to. If*]

**Response:** We have referred ‘to the latest version of the Declaration of Helsinki’ and added such a description (p. 7, line 129).

**5. Comment:** [*Line 147, was the right foot also the dominant foot of all participants?*]

**Response:** We appreciate the **editor**'s question. The right foot was also the dominant foot of all participants (p. 7, lines 127-128).

**6. Comment:** [*Line 161: if there are 12 trials during the pre-test, does it mean that there were 4 trials for each of the three speeds? Please clarify. Likewise, in line 177, why do you calcualte the average of all 12 trials and not separately per speed? See also another comment about the justification to use different speeds.*]

**Response: We understand your point and agree with you. Following the editor’s suggestion, we have clarified the procedure** (p. 9, lines 192–193) and justified the adoption of different speeds (p. 8, lines 157-161).

**7. Comment:** [*Line 161: is each of the four blocks devoted to one of the four directions? If the directions were 'blocked', please explain why.*]

**Response:** We have revised our **description** **to be more clearly understood** (p. 9, lines 195–196), showing that each block consisted of three different speeds and four different directions.

**8. Comment:** [*Line 180 and 182 (possibly also elsewhere): please specify what does a certain direction of effect mean. For instance, when the difference is 'smaller' (page 180), does it mean that the error was smaller in the pre- or in the post-test? Related to this, the figures show both negative and positive values for AE and VE. Please explain what does the sign of each value mean. The same comment applies to the other variables that have values of two signs (e.g., horizontal and saggital head angle, etc.).* ]

**Response: We agree with you and** have specified the meaning of a certain direction of effect regarding each variable in the *Methods* (p. 11, lines 222-229), along with explaining the meaning of sign of each value (p. 14, lines 279-281) as well as the other variables (p. 14, lines 302-308).

**9. Comment:** [*Line 196: here you describe an effect but the direction is unclear. I recommend specifying in the text which group had a larger VE. This applies to other related instances.*]

**Response:** We appreciate the editor's helpful suggestions in pointing out the description of the effect. In our revisions, we have modified the result of VE accordingly (p.12, lines 243-245).

**10. Comment:** [*Lines 234ff: this paragraph can be written in a more concise way as there are several similar text passages that are being repeated. Perhaps you could combine the descirption about the absence of some effects in single sentences (e.g., head angle in horizontal plane in near and far conditions was not affected by group and this can be mentioned in a single sentence with both statistical results in brackets).* ]

**Response:** We understand your point and agree with you. We have revised this paragraph by making it more concise, combining the various descriptions of the absence of some effects in single sentences (p.14, lines 285-296).

**Comments from Reviewer #1:**

**General Comment:** *In general, the work seems well-written and detailed. I only have a few comments. However, I want to inform the authors that I am not an expert in the field.*

**Response:** We wish to express our strong appreciation to the reviewer for their constructive and insightful comments on our paper. The comments have helped us significantly improve the paper. We have carefully considered the reviewer’s comments and incorporated changes that reflect the detailed suggestions you have generously provided.

Please see below our detailed response to each comment where we also describe the changes made to the manuscript. All page numbers refer to the revised manuscript file with tracked changes.

**1. Comment:** [*The introduction is clearly written, however, in my opinion, it would be useful to distinguish between head movements used to orient towards a direction (e.g., towards the ball) and head movements that are not necessarily directed towards the ball but serve to inspect the acoustic space and increase the possibility of extracting acoustic cues (e.g., rotations to move the cone of confusion...). See for instance Valzolgher et al., (2022) in which the head-movements were used strategically to localize sounds sources.*]

**Response:** We appreciate the reviewer’s instructive suggestion. We agree with you and have modified the introduction accordingly (p.3, lines 44-49). We have added a paragraph and presented the distinction between the head movements that are not necessarily directed towards the ball but serve to inspect the acoustic space and increase the possibility of extracting acoustic cues and the head movements that are used to orient towards a direction.

**2. Comment:** [*The methods section lacks a discussion on how the sample size was determined. 10 vs 10 participants do not seem to be many; are there previous studies that justify this sample size?*]

**Response:** We appreciate the reviewer’s helpful suggestion. Accordingly, we have added a description to the *Methods* (p. 6, lines 122-124) to justify the sample size adopted in our study based on previous studies. Thus, we believe that it is possible to retain our results.

**3. Comment:** [*Line 90: Did the participants play football? I understand they had no experience with blind football, but did they have experience with regular football?*]

**Response:** We appreciate your question. The enrolled participants had no previous regular football training. We have added this description in the *Methods* (p. 6, lines 118).

**4. Comment:** [*Line 180: Smaller than what?*]

**Response:** We appreciate the reviewer's comment. Following the reviewer’s suggestion, we have modified the description in the *Methods* (p.11, lines 222-223) to explain that the error decreased from the pre- to post-test.

**5. Comment:** [*Line 182: More downward than what?*]

**Response:** Following the reviewer’s suggestion, we have modified the description in the *Methods* (p. 11, lines 223-229) to explain that the head and trunk showed a greater downward rotation from the pre- to post-test. Moreover, to clarify the explanation of the head–trunk rotation angle in the sagittal plane, we have now also explained that a negative value for the amount of difference indicated that the head was rotated more downward in relation to the trunk from the pre- to post-test.

**6. Comment:** [*Figure 5: Why are there 6 lines per group? I understood there were 10 vs 10 participants. Was there some filtering?*]

**Response: We apologise for the confusion regarding the number of lines per group.** **Following the reviewer’s comments, we have revised Figure 5** **to be more clearly understood, now showing 10 lines per group.**

**7. Comment:** [*Both in the introduction and discussion, the authors could consider adding references to the literature on motor training aimed at improving sound localization involving head movement. This could also help better describe the head strategies used in this study.*

*Valzolgher, C., Campus, C., Rabini, G., Gori, M., & Pavani, F. (2020). Updating spatial hearing abilities through multisensory and motor cues. Cognition, 204, 104409.*

*Valzolgher, C., Todeschini, M., Verdelet, G., Gatel, J., Salemme, R., Gaveau, V., ... & Pavani, F. (2022). Adapting to altered auditory cues: Generalization from manual reaching to head pointing. PLoS One, 17(4), e0263509.*]

**Response:** We thank you for providing these insights. The reviewer’s suggestion has helped us improve our arguments and describe the head strategies used in this study. We have added the point describing the role of head movements for improving sound localization during spatial training (p. 3, lines 44-49). We have also discussed the effectiveness of multisensory-motor training aimed at improving sound localization involving head movements according to the reviewer’s suggestion (p. 23, lines 428-435).

**8. Comment:** [*Page 375: Regarding, this study might be of interest: Rabini, G., Altobelli, E., & Pavani, F. (2019). Interactions between egocentric and allocentric spatial coding of sounds revealed by a multisensory learning paradigm. Scientific reports, 9(1), 7892.*]

**Response:** We appreciate the reviewer’s point regarding the egocentric-allocentric issue. We have added the suggested content in the *Discussion* (p. 25, lines 465-471).

**Additional clarifications**

In addition to the above comments, all grammatical errors have been corrected. The references have also been formatted per journal guidelines.
