# Supplementary material for: Head movements affect skill acquisition for ball trapping in blind football
Source: PLoS One. 2024 Nov 7;19(11):e0313336. doi: 10.1371/journal.pone.0313336 (PMC11542860; doi:10.1371/journal.pone.0313336)
Supplement: Attachment — Submitted filename: Response_to_Reviewers.docx [file pone.0313336.s004.docx]

**Comments from** **Editor:**

**General Comment:** *Thank you for submitting your manuscript to PLOS ONE. After careful consideration, we feel that it has merit but does not fully meet PLOS ONE’s publication criteria as it currently stands. Therefore, we invite you to submit a revised version of the manuscript that addresses the points raised during the review process by reviewer #2.*

**Response:**

We wish to express our sincere appreciation to the Editor for the constructive comments on our paper. These comments have significantly improved the manuscript. We have carefully considered the Editor’s feedback and incorporated changes that address the points generously provided by Reviewer #2.

Please see below our detailed response to each comment, where we also describe the changes made to the manuscript. All page numbers refer to the revised manuscript file with tracked changes.

**Comments from Reviewer #2:**

**General Comment:** *Congratulations, the topic of the paper is interesting. The manuscript is well written, the content is clearly presented, as are the results, figures and tables. Considering the corrections and changes made by the authors, following the comments of the other reviewers, this study has improved the construction of the hypothesis, and results' clarity, understanding. The Authors investigated the incidence of head movements during the acquisition of the ball trapping task in blind football. The study provides important insights that could help the training methods of bling football, especially during the learning of ball location in space by beginners. The specific hypothesis is well formulated, and the rationale is consistent with the current literature. However, not being an expert in the field, I would like to ask you for some clarification on the methods and I have some minor comments that, I think, can be addressed to improve the understanding of the article.*

**Response:** We wish to express our sincere appreciation to the reviewer for the constructive and insightful comments on our paper. These comments have significantly improve the manuscript. We have carefully considered the reviewer’s comments, especially regarding clarifications on the methods, and have incorporated changes that reflect the detailed suggestions you have generously provided.

Please see below our detailed response to each comment, where we also describe the changes made to the manuscript. All page numbers refer to the revised manuscript file with tracked changes.

**1. Comment:** *In the procedure section, what was the approximate duration of each day of skill acquisition training, did the duration vary for the two groups and between participants within each group? If there were differences, please specify in the manuscript text.*

**Response:** **We appreciate the reviewer's comment. We have added a description of the** **duration of skill acquisition training on both day 1 and day 2 (p10, lines 209–211).**

**2. Comment:** *Also in the procedure section: how much time elapsed between skill acquisition and pre-test on day 1, and between post-test and acquisition on day 2? In addition, if possible I would ask to specify whether the two days, which the procedure refers to, were consecutive or non-consecutive.*

**Response: Thank you for your insightful question. In response,** **we have clarified that there was a 15-min interval between the pre-test and skill acquisition phase on day 1, with approximately 8 min spent explaining the instructions. On day 2, there was a 7-min interval between the skill acquisition phase and the post-test (p9, lines 193–197). We also confirm that the experiment was conducted on consecutive days for all participants (p10, lines 198–199).**

**3. Comment:** *In the apparatus section, when describing the experimental set-up, I suggest specifying in the manuscript the distance between the apparatus for throwing the ball andthe participants (although shown in figure.1) Since it represents an important data in the structure of the protocol, as the distance could influence the final speed of the ball and the orientation of the participants' head.*

**Response:** We appreciate the reviewer’s instructive suggestion. We agree and have added a description of the distance between the launching point of the ball and the reference point where the participants stood (p.8, lines 156–157).

**4. Comment:** *From the 158 to the 161, the period is not apparent to me, it seems almost confusing. Defined that speed is not a dependent variable of your study. I cannot understand if it is indicated that* *a randomization of the velocities was made* *to prevent the learning effect of the subjects, or that the velocities were not randomized because it was not a variable of study interest. In both cases, the authors could clarify the sentence.*

**Response:** We apologize for the confusion regarding the randomization of the velocities. In response, we have revised the description in the Methods section (p. 8, lines 158–162) to clarify the randomization.

**5. Comment:** *Were the combinations of speed and trajectory* *of the ball thrown administered in a randomized order, with a pre-defined order for all participants, or was it chosen independently by the experimenter who threw the ball? Was the experimenter who moved the device always the same in all tests? Please clarify and specify these aspects if possible.*

**Response:** We appreciate your question. The combinations of ball speed and trajectory were administered in a randomized order, following a predefined order for all participants. Furthermore, the same experimenter moved the device to ensure consistency and control for any variability. We have added these descriptions in the Methods section (p.8, lines 168–170).

**6. Comment:** *At line 193 it is not clear whether the familiarization tests were carried out at a fixed speed and direction, or also for the 12 familiarization tests they represent the combination of heights and speeds? If possible, better specify the concept.*

**Response:** We appreciate the reviewer's comment. In response, we have revised the description in the *Methods* section (p.10, lines 203–204) to clarify that the combinations of ball speed and trajectory were administered in a pre-defined randomized order during the practice trials.

**7. Comment:** *Line 194 when reporting of the 48 tests (12 tests 4 blocks) during the acquisition of the ball catching ability, the authors mean that the 12 combinations (3 directions x 4 heights) were repeated 4 times. If so, were the 4 blocks all administered in the same order or were they randomly ordered? Please* *clarify the concept.*

**Response:** Following the reviewer’s suggestion, we have revised the description in the *Methods section* (p. 10, lines 207–208) to clarify that the 4 blocks were administered in a randomly determined order.

**Additional clarifications**

In addition to the above comments, all grammatical errors have been corrected. The references have also been formatted per journal guidelines.
